# Analysis on Biofertilization-Induced Memory Acquisition for Heat Stress Mitigation in Soybean Plants

**DOI:** 10.3390/plants15101468

**Published:** 2026-05-12

**Authors:** Helena Chaves Tasca, Douglas Antônio Posso, Eugenia Jacira Bolacel Braga, Elise Réthoré, Sylvain Pluchon, Giancarlo Ribas Valduga, João Paulo Smith, Luiz Fernando Melgaço Bloisi, Gustavo Maia Souza

**Affiliations:** 1 Plant Cognition and Electrophysiology Laboratory, Federal University of Pelotas, Pelotas 96160-000, Brazil; hctasca@gmail.com (H.C.T.); gumaia.gms@gmail.com (G.M.S.); 2 Plant Tissue Culture Laboratory, Federal University of Pelotas, Pelotas 96160-000, Brazil; jacirabraga@hotmail.com; 3 Centre Mondial de l’Innovation Roullier, Groupe Roullier, 35400 Saint-Malo, France; elise.rethore@roullier.com (E.R.); sylvain.pluchon@roullier.com (S.P.); 4 TIMAC Agro Brasil, Groupe Roullier, Porto Alegre 90480-000, Brazil; giancarlo.valduga@timacagro.com.br (G.R.V.); joao.smith@timacagro.com.br (J.P.S.); luiz.bloisi@timacagro.com.br (L.F.M.B.)

**Keywords:** antioxidant metabolism, photosystem II efficiency, redox homeostasis, stomatal conductance, stress priming

## Abstract

The increasing frequency of high-temperature episodes associated with climate change poses challenges to crop productivity. Stress priming could help to mitigate these effects, with the capacity to enhance plant resilience through metabolic adjustments and memory mechanisms. We evaluated the efficacy of the Stress Memory Encoder biofertilizer (SME, TIMAC Agro) as a seed treatment to induce heat stress (HS) memory in soybean plants [*Glycine max* (L.) Merrill]. In Experiment 1, plants with SME (0, 2, and 4 mL kg^−1^) were exposed to HS (35 °C for 48 h) at V3 and V6 vegetative stages. The 4 mL kg^−1^ dose at V6 under HS consistently improved photosynthetic traits and reductions in reactive oxygen species and lipid peroxidation. Non-enzymatic antioxidants were detected to this dose at V3. Multivariate analysis revealed patterns consistent with dose-dependent physiological adjustments and potential memory acquisition. In Experiment 2, plants treated with SME were exposed to HS (34 °C for 48 h) consecutively (V3 + V6). The SME-primed plants had a higher expression of transcript factors and genes related to HS. Overall, the findings indicate that SME may act as a priming agent capable of inducing somatic memory and enhancing adaptive responses to HS in soybean.

## 1. Introduction

Plants, being sessile organisms, are continuously exposed to environmental fluctuations that can compromise their development and productivity. These challenges include variations in temperature, light intensity, water, nutrient availability, pathogens, and herbivory. To cope with such stresses, plants rely on highly integrated mechanisms involving metabolic regulation, antioxidant systems, signaling cascades, and gene expression networks that modulate responses to external cues [1,2,3].

Among the abiotic factors, heat and drought are particularly detrimental and are currently the main constraints to global agricultural performance [4,5]. These stressors affect key physiological processes such as photosynthesis and transpiration, impair nutrient assimilation [6,7], and promote the generation of reactive oxygen species (ROS), which damage cell membranes and photosynthetic structures, which triggers antioxidant responses [8,9,10].

In the context of recurrent and increasingly intense environmental stress, the reason why we performed this study is that plants that are able to “remember” previous exposure events, such as mild stress early on in plant development or by seed treatment application, leading them to respond more efficiently to subsequent ones, especially stress factors during its cultivation, which cause them to gain an adaptive advantage [11,12]. This ability, often referred to as priming, hardening, or conditioning, results in a form of stress memory [2,13]. In primed plants, an initial mild stimulus prepares physiological and molecular systems to react more robustly and rapidly when the stress recurs [14,15,16]. Priming can be induced through biotic or abiotic stimuli, or via exogenous application of chemical compounds such as jasmonates, salicylic acid, and biostimulant [17]. After the priming event, plants may retain biological information (memory of the stress), altering their physiological or metabolic responses upon re-exposure [16]. Somatic memory stress, typically transient and restricted to the individual plant, is thought to involve epigenetic mechanisms, including chromatin remodeling and RNA interference [16,18,19].

In recent years, the literature has documented the presence of stress memory in plants, highlighting its relevance for crop improvement and stress mitigation [11,20,21,22]. While yield remains a central focus in breeding programs, enhancing tolerance to environmental stressors is equally critical. Accordingly, seed priming with biostimulants, products composed of natural or synthetic compounds capable of modulating plant metabolism, has emerged as a promising tool to improve stress resilience [23,24]. In this context, studies regarding seed treatment with products capable of memory inducing, or priming effects, can improve field cultivation and make plants more resilient when exposed to stress factors during their growth and production; if they do not improve a plant’s yield, they at least reduce the loss caused by stress factors that can cause loss in productivity, such as heat stress.

Biostimulant may act through diverse biochemical pathways to regulate hormonal balance, stimulate root development, and increase tolerance to abiotic stress [25,26]. Some of these compounds promote water and nutrient uptake and are known to mitigate damage caused by drought by increasing radicular system and hormonal homeostase [25]. However, plant responses vary according to species, stress type, and biostimulant composition, underscoring the need for empirical validation [27]. According to these presumptions about biofertilizers, we are trying to prove that there is a possibility for somatic memory encoding when utilizing specifical commercial products for seed treatment.

Within this framework, the present study investigates the potential of a biofertilizer, Stress Memory Encoder (SME, a commercial product from TIMAC Agro Industry and Commerce of Fertilizers, Porto Alegre, RS, Brazil), using two doses to promote heat stress memory in soybean plants. Specifically, we assessed whether seed priming with SME improves physiological and biochemical performance following exposure to high temperatures during plant growth, at two different vegetative stages. The main reason for this is that we hypothesize that the Stress Memory Encoder (SME) biofertilizer induces heat stress-related somatic memory in soybean plants, with effects that persist throughout the crop cycle even when the product is applied exclusively as a seed treatment prior to germination, as a priming. We specifically tested whether SME-mediated priming establishes a durable stress memory by exposing plants to heat stress at two distinct developmental stages, thereby enabling discrimination between short- and long-term memory effects. Furthermore, we assessed whether this induced memory enhances plant resilience, as evidenced by plants’ physiological and molecular behavior under heat stress conditions.

## 2. Materials and Methods

### 2.1. Experiment 1, Physiologycal and Biochemical Behavior

#### 2.1.1. Environmental Conditions and Plant Material

To develop this research, the Stress Memory Encoder biofertilizer (SME, a commercial product from TIMAC Agro Industry and Commerce of Fertilizers, registered in the Brazilian Ministry of Agriculture and Livestock under registration number RS 000155-0.000273), was used. In Brazilian legislation, biofertilizer means “a product containing an active ingredient or organic agent, free of agrotoxic substances, capable of acting, directly or indirectly, on all or part of cultivated plants, increasing their productivity, without taking into account its hormonal or stimulant value” [28]. In the case of SME, it is extracted from marine algae at a plant factory.

For this, an experiment was carried out under controlled greenhouse conditions between July and September 2023. The trials employed soybean seeds [*Glycine max* (L.) Merrill] cultivar Brasmax Valente RR, subjected to different concentrations of the biofertilizer and to a high-temperature condition in two different growing stages independently. The soybean cultivar Brasmax Valente RR (Maturity Group 6.7) is a Roundup Ready^®^ genotype with an indeterminate growth habit, high branching capacity, and good lodging resistance. It shows broad adaptation to different environments, stable agronomic performance, and resistance to major diseases such as stem canker, Phytophthora root rot, and frogeye leaf spot.

Seeds were treated with SME at 0, 2 and 4 mL seeds kg^−1^. The seeds, contained in a plastic bag, were mixed with the respective SME treatments until a homogeneous film was formed on them. After treatment, seeds were sown in 1 L plastic pots (perforated at the base for drainage) filled with a standardized commercial substrate Beifort^®^ S-10B (composed of organic waste from seeds, pomace and grape stalks, peat and carbonized rice husks, pH 6.8 ± 1.0, conductivity 1.0 mS cm^−1^, density 350 kg m^−3^, water retention capacity of 70.0%). Five seeds were initially sown per pot, and after thinning, two seedlings were maintained through the entire experiment per pot: one intended for morphological analysis, the other for biochemical analysis in each pot; we have used four pots/replicates for morphological and biochemical analysis.

Environmental conditions were maintained at 25 ± 2 °C and 70% relative humidity at greenhouse conditions. Irrigation was performed every two days, alternating between distilled water and Hoagland and Arnon’s nutrient solution [29], until the plants reached the targeted phenological stages V3 (vegetative growing stage with two trifoliate leaves present) or V6 (vegetative growing stage with five trifoliate leaves present) as described by Fehr & Caviness [30].

**Figure 1:**
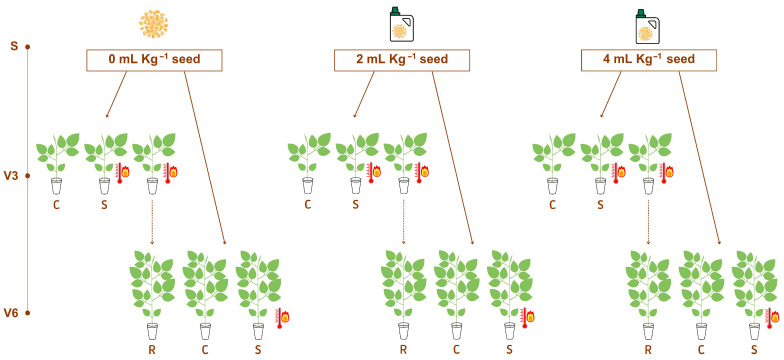
Experimental design of the experiment with the 2023 SME Formulation to test biofertilizer doses in soybean [*Glycine max* (L.) Merr., cv. Brasmax Valente RR] under high temperature conditions at V3 and V6 stages (R = recovery, C = control, S = stress).

To impose heat stress treatment, a group of plants at V3 stage were subjected to a high-temperature stress condition of 35 °C for 48 h in a climate-controlled growth chamber (70% relative humidity, and 750 µMol m^−2^ s^−1^ light irradiance) to test a short-time priming effect of the SME biofertilizer. When this first group entered the growth chamber, the control group (C) was also transferred to a second growth chamber, in control conditions (25 °C, 70% relative humidity, and 750 µMol m^−2^ s^−1^ light irradiance) and stayed inside this control chamber until the end of the experiment. Following the stress treatment at V3 stage, a group of plants was allowed to recover under control conditions until reaching V6 (recovery group from V3 stress imposing, R Group, at control chamber). When a second group of plants reached the V6 stage, the same heat stress condition was applied on this naïve group of plants in order to test the eventual long-time priming effect of the SME biofertilizer (Figure 1).

#### 2.1.2. Physiological Assessments

Physiological parameters were assessed in the most recently fully expanded leaf. Stomatal conductance, Transpiration Rate, humidity of the leaf, temperature of the leaf, and vapor pressure deficit of the leaf (VPD) were measured using a porometer/fluorometer system (LI-600, LI-COR^®^, Lincoln, NE, USA), and chlorophyll and fluorescence (ΦPSII) was recorded under growth chamber light conditions. Chlorophyll content was determined using a ClorofiLOG^®^ CFL 1030 m (Falker, Porto alegre, RS, Brazil). All measurements were conducted in triplicate per plant.

#### 2.1.3. Biochemical Analysis

Hydrogen Peroxide (H_2_O_2_) was quantified using a microplate-based method [31], with technical duplicates. Each well received 50 µL of enzyme extract, 50 µL of potassium iodide (1 M), and 100 µL of potassium phosphate buffer (pH 7.0). Absorbance was read at 390 nm and concentrations were calculated using a correction factor of 0.54.

Superoxide (O_2_^−^) generation rate was determined as described by Chaitanya & Naithani [32], using a reaction mixture containing 100 mM sodium phosphate buffer (pH 7.2), 1 mM sodium diethyldithiocarbamate, and 0.25 mM nitro blue tetrazolium (NBT). A 100 µL aliquot of enzyme extract was added. Absorbance was read at 540 nm every 20 s for 3 min. Final O_2_^−^ concentration was calculated as the difference between the last and first readings.

Lipid peroxidation was estimated via Malondialdehyde (MDA) content [33], a proxy for membrane damage and reactive substances with thiobarbituric acid (TBARS). Tubes containing 400 µL of enzyme extract, 100 µL of 10% trichloroacetic acid, and 500 µL of 0.67% TBA were sealed, incubated at 100 °C for 15 min, cooled, and centrifuged at 2000 rpm (1000× *g*) for 15 min. Supernatant absorbance was read at 535 nm, and MDA concentration calculated using a standard curve.

Anthocyanins were extracted from ~100 mg of fresh leaf tissue in 2 mL of methanol:HCl (99:1, *v*/*v*) at 4 °C for 24 h [34]. Chlorophylls were removed by adding 1.5 mL chloroform and 3 mL deionized water. Anthocyanin content was calculated as cyanidin-3-glucoside equivalents (MW = 449.2 g mol^−1^, ε530 = 34,300 L mol^−1^ cm^−1^), expressed as mg 100 g^−1^ FW.

Phenolic and flavonoid contents were assessed using aliquots from the same extract above, read at 280 and 325 nm with methanol:HCl and water as blank. Total phenolics and Flavonoids were calculated following Fukumoto & Mazza [35], expressed as A280 g^−1^ FW and A325 g^−1^ FW.

#### 2.1.4. Statistical Analysis for Experiment 1

The experiments followed a completely randomized design with 4 biological replicates per treatment and sampling point. Functional trait variation among biofertilizer doses, as well as the phenological stage at which stress was applied, were analyzed using Two-way ANOVA, and when F was significant followed by Tukey’s HSD post hoc test with the significance set at *p* ≤ 0.05.

For the VPD, leaf temperature, and leaf humidity, the treatments were analyzed using One-way Anova, and when F was significant followed by Tukey’s HSD post hoc test with the significance set at *p* ≤ 0.05.

Principal Component Analysis (PCA) with the 10 most significant loading variables was done, and the heatmap correlates the results with color gradients representing the Z-score (standardized values), ranging from blue (downregulation/decrease) to red (upregulation/increase). The dendrograms indicate the similarity between treatments (top) and variables (left) based on Euclidean distance. All statistical analyses were performed using R version 4.2.0 (R Core Team, Wien, Austria), with significance set at *p* ≤ 0.05.

### 2.2. Experiment 2, Gene Expression Analysis

#### 2.2.1. Plant Material, and Growth Condition

An experiment was performed to determine the effect of SME biofertilizer on the gene expression of transcription factors and proteins involved in heat stress tolerance after two consecutive short stresses. Soybean seeds [*Glycine max* (L.) Merrill] cv RGT Symbala: this is a non-GMO European genotype developed by RAGT, characterized by an indeterminate growth habit and balanced plant architecture, supporting stable canopy development and branching. It is adapted to temperate environments and integrated cropping systems, with breeding emphasis on yield stability, protein content (~40%), and efficient nitrogen use typical of modern European cultivars.

The seeds were imbibed in water or in the SME biofertilizer diluted at 7% for 2 h, then rinsed with distilled water and sowed in trays. Three days after sowing, each plant was inoculated with 3 mL of *Bradyrhizobium japonicum* inoculum at 10^8^ UFC mL^−1^. After ten days, the seedlings were transferred to 4 L pots filled with a standardized commercial substrate (1 plant/pot). Fifteen days after transplanting (stage V3), five plants (Control) were kept at 24 °C/18 °C while the other plants were transferred at 34 °C/28 °C for 48 h. Then the plants were transferred back to 24 °C/18 °C for 11 days before a second heat stress for 48 h (stage V5–V6).

#### 2.2.2. Gene Expression Analysis

After the second stress, the aerial parts of the plants were frozen in liquid nitrogen and were ground to a fine powder in the presence of liquid nitrogen, and the total RNA was extracted using a Nucleospin 8 RNA kit (Macherey-Nagel, Düren, Germany) following the protocol of the manufacturer. The quality and yield of all RNA samples were analyzed and checked in a 4200 Tapestation (Agilent Technologies, Santa Clara, CA, USA) followed by DNase treatment and complementary DNA (cDNA) synthesis from 1 µg RNA using an iScript genomic DNA (gDNA) clear cDNA synthesis kit (Bio-Rad, Hercules, CA, USA). Quantitative RT-PCR (qPCR) analysis was performed in a total volume of 10 µL using the Universal SYBR Green Supermix (Bio-Rad) in an RT PCR Detection System (Bio-Rad). The qPCR reactions were performed in technical triplicates using independent cDNA reactions for each biological replicate and 300 nM gene-specific primer pairs. Specific primers for all candidate genes (*GmHsf25*, *GmHSf34*, *GmDREB2-A2*, *GmHSP90A1*, *GmHSP90A2*, *GmHSP90B1* and *GmHSP90C2.1*) were designed using Primer3 software (version 0.4.0). The thermal cycler protocol included 98 °C for 3 min, 40 cycles of 98 °C for 15 s, 60 °C for 30 s, 72 °C for 15 s, and a final 3 min extension at 72 °C. The expression of all the candidate genes was normalized against four soybean reference genes, namely *GmACTIN1*, *GmEF1alpha*, *GmTUBULIN* and *Gm18srRNA*. The stability of these reference genes across all samples was assessed using the Reference Gene Selection tool integrated in CFX Maestro software version 2.3 (Bio-Rad, Hercules, CA, USA). Normalized expression levels of selected candidate genes for each sample were subsequently calculated using CFX Maestro version 2.3 (Bio-Rad) using the method of relative normalized gene expression ΔΔCt [36,37].

Primer sequences (5′–3′) used for RT–qPCR amplification were as follows: *GmACTIN11* (F: GTGTTGGATTCTGGTGATGGTG; R: GGCTGAGGTGGTGAAGGAA), *GmEF1α* (F: GGCTGATTGTGCTGTCCTT; R: TCACGAGTCTGTCCATCCTT), *GmTUBULIN* (F: AGAACTGCGACTGCCTCC; R: CAAAAGCGTCCCCATCCC), *Gm18S rRNA* (F: AACCATAAACGATGCCGACC; R: TTTCAGCCTTGCGACCATAC), *GmHsf25* (F: TCAACCTCGTCCTCGTCC; R: TTCAGTTTCTCGTTCTCGCTC), *GmHsf34* (F: GAAGGTGAGTTGGAGAGGTTG; R: GAGTTCAGTTGTTGGTGCCT), *GmHsp90A1* (F: GGACCTTGTGTTGCTTCTCTT; R: ATCTCCGCCATTGTCATCCT), *GmHsp90A2* (F: TCAGCCACAAAGGAAGGGT; R: AGTCACCAAACAGCAAGGAG), *GmHsp90B1* (F: TCCGAGAGAGAGTAGTAAAGAACC; R: CCCATTTGAGTTGAGGAAAGAGA), *GmHsp90C2.1* (F: ACGATACAACAACTGAAGGCA; R: TCCCAATACCGCTCAACAAC), and *GmDREB2A;2* (F: AAAGTAGGAGCAGAGGGTATGG; R: GCCAGAATAAAGGTGTTCGTTG).

#### 2.2.3. Statistical Analysis for Experiment 2

This experiment followed a completely randomized design with 5 biological replicates per treatment. For diffrentrial gene expression the data were analyzed using One-way ANOVA, and when F was significant followed by Tukey’s HSD post hoc test with the significance set at *p* ≤ 0.05.

## 3. Results

### 3.1. Experiment 1, Physiologycal and Biochemical

#### 3.1.1. Physiological Assessments

**Figure 2:**
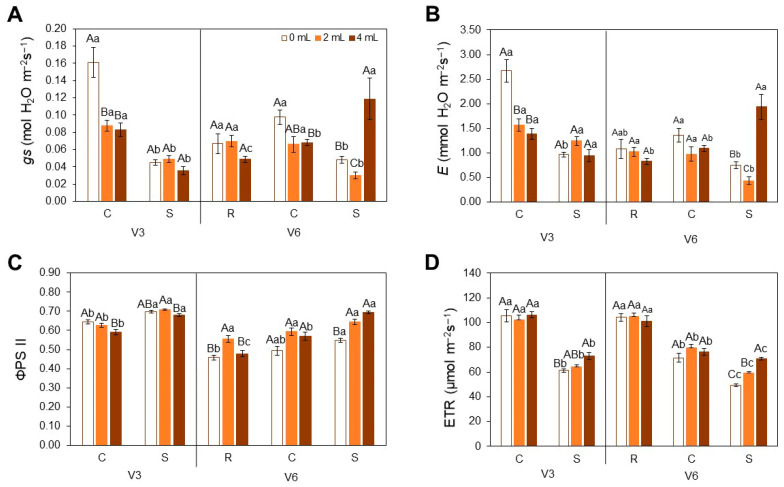
Stomatal conductance (*g_s_*) (**A**), Transpiration Rate (*E*) (**B**), Photosystem II Quantum Efficiency (ΦPSII) (**C**) and Electron Transport Rate (ETR) (**D**) in soybean [*Glycine max* (L.) Merr., cv. Brasmax Valente] under high temperature conditions at V3 and V6 stages. Bars represent means ± standard error (n = 4). Capital letters indicate significant differences among SME doses (0, 2 and 4 mL kg^−1^ seed) within each heat treatment condition (R = recovery, C = control, S = stress). Lowercase letters indicate significant differences between heat treatment conditions (CxS in V3 and RxCxS in V6) for each dose. Statistical comparisons were performed using Two-way ANOVA followed by Tukey’s post hoc test (*p* ≤ 0.05).

In the gas exchange parameters (Figure 2), plants at V3 under control conditions (C) and at the 0 mL dose exhibited the highest stomatal conductance (*g_s_*) and transpiration (*E*), significantly surpassing the values observed for 2 and 4 mL (Figure 2A,B). Under heat stress (S), both *g_s_* and *E* declined across all doses. At V6, no significant differences were found among doses under recovery conditions (R), although the 4 mL dose showed the lowest mean values. Under S, the 4 mL dose promoted higher *g_s_* and *E* values.

Regarding Photosystem II Quantum Efficiency (ΦPSII) (Figure 2C), this increased under S at V3, but the 4 mL dose consistently showed the lowest values. At V6, the lowest ΦPSII values occurred in the 0 mL treatment under all conditions. Notably, ΦPSII increased significantly at the 2 mL dose under R, and a trend of gradual improvement was observed under S as biofertilizer dose increased. For Electron Transport Rate (ETR) (Figure 2D), a reduction was observed under S at V3, but ETR increased in parallel with the biofertilizer dose. At V6, the highest ETR was detected under R.

**Figure 3:**
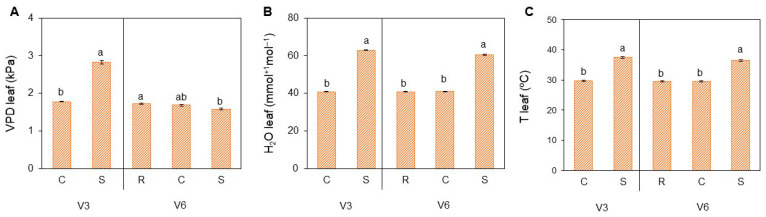
Vapor pressure deficit (VPD) (**A**), leaf humidity (**B**), and leaf temperature (**C**) during porometer/fluorometer analysis in soybean [*Glycine max* (L.) Merr., cv. Brasmax Valente] under high temperature conditions at V3 and V6 stages. Bars represent means ± standard error (n = 4). Letters indicate significant differences between heat treatment conditions (CxS in V3 and RxCxS in V6), according to One-way ANOVA followed by Tukey’s post hoc test (*p* ≤ 0.05).

In an attempt to better explore the data obtained by the porometer/fluorometer, we show in Figure 3 the results that are presented during analysis. It is evident that stress conditions at V3 led to significantly higher vapor pressure deficit (VPD) (Figure 3A), leaf humidity (Figure 3B), and leaf temperature (Figure 3C). During the S at V6 stage, the plants presented the same pattern before, with the exception of VPD, which showed results that were not as high as in V3 at the leaf surface.

**Figure 4:**
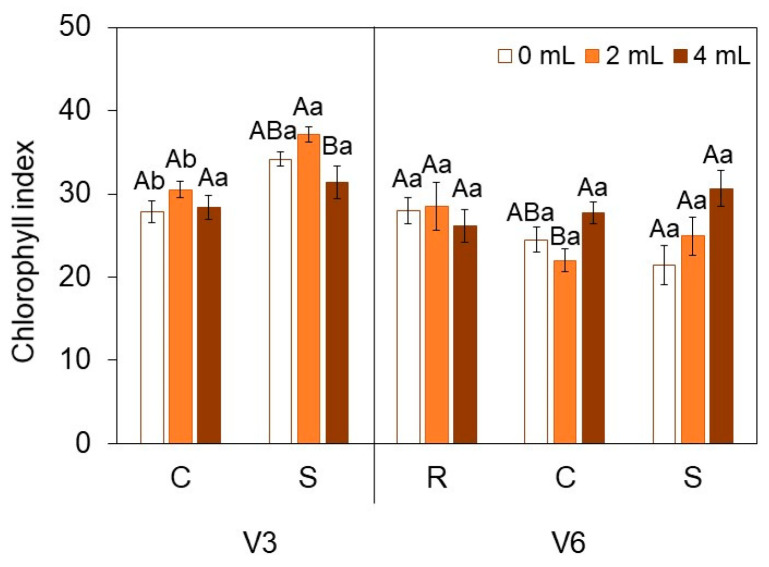
Chlorophyll index in soybean [*Glycine max* (L.) Merr., cv. Brasmax Valente] under high temperature conditions at V3 and V6 stages. Bars represent means ± standard error (n = 4). Capital letters indicate significant differences among SME doses (0, 2 and 4 mL kg^−1^ seed) within each heat treatment condition (R = recovery, C = control, S = stress). Lowercase letters indicate significant differences between heat treatment conditions (CxS in V3 and RxCxS in V6) for each dose. Statistical comparisons were performed using Two-way ANOVA followed by Tukey’s post hoc test (*p* ≤ 0.05).

In chlorophyll index analyses (Figure 4), the 4 mL dose showed a significant reduction under S at V3 when compared to 2 mL. At V6, the 2 mL dose under C presented lower values than the 4 mL dose. Under S, a dose-dependent increase in chlorophyll index was observed.

#### 3.1.2. Biochemical Assessments

**Figure 5:**
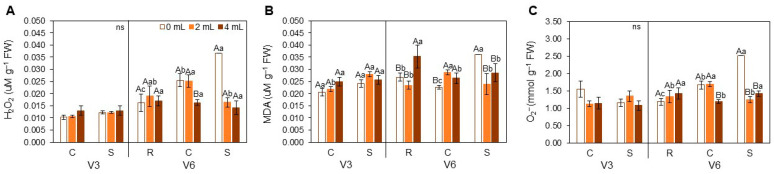
Hydrogen Peroxide (H_2_O_2_) (**A**), Malondialdehyde (MDA) (**B**) and Superoxide generation rate (**C**) (O_2_^−^) in soybean leaves [*Glycine max* (L.) Merr., cv. Brasmax Valente] under high temperature conditions at V3 and V6 stages. Bars represent means ± standard error (n = 4). Capital letters indicate significant differences among SME doses (0, 2 and 4 mL kg^−1^ seed) within each heat treatment condition (R = recovery, C = control, S = stress). Lowercase letters indicate significant differences between heat treatment conditions (CxS in V3 and RxCxS in V6) for each dose. ns = non-significant data. Statistical comparisons were performed using Two-way ANOVA followed by Tukey’s post hoc test (*p* ≤ 0.05).

Biochemical assays showed no significant differences in Hydrogen Peroxide (H_2_O_2_) at V3 (Figure 5A). At V6, the 4 mL dose under C had the lowest H_2_O_2_ content. Under S, H_2_O_2_ was highest at 0 mL, followed by C and then R.

Malondialdehyde (MDA) (Figure 5B) levels increased at 4 mL under R at V6 and were lowest at 0 mL under C. Under S, the highest MDA values were observed at 0 mL. The limited MDA variation indicates that oxidative damage was minimal and that antioxidant systems were efficient in mitigating lipid peroxidation.

For Superoxide (O_2_^−^) (Figure 5C), no significant differences were found at V3. At V6, nearly all environmental comparisons differed, except the 4 mL dose under R and S, which showed similar values. Notably, under S, the 0 mL dose had the highest O_2_^−^ concentrations, while the 2 and 4 mL doses showed reductions, suggesting improved ROS scavenging.

**Figure 6:**
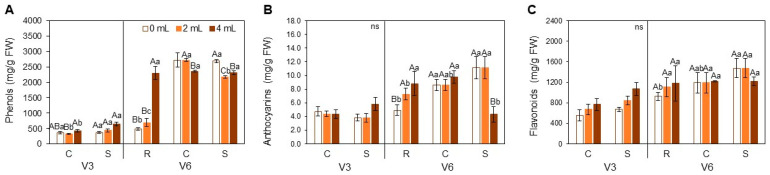
Phenols (**A**), Anthocyanins (**B**) and Flavonoids (**C**) in soybean leaves [*Glycine max* (L.) Merr., cv. Brasmax Valente] under high temperature conditions at V3 and V6 stages. Bars represent means ± standard error (n = 4). Capital letters indicate significant differences among SME doses (0, 2 and 4 mL kg^−1^ seed) within each heat treatment condition (R = recovery, C = control, S = stress). Lowercase letters indicate significant differences between heat treatment conditions (CxS in V3 and RxCxS in V6) for each dose. ns = non-significant data. Statistical comparisons were performed using Two-way ANOVA followed by Tukey’s post hoc test (*p* ≤ 0.05).

Non-enzymatic antioxidants also varied across treatments. At V3, phenolic content was significantly higher at 4 mL compared to 2 mL under C (Figure 6A). Under S, phenolics increased in SME treatments. At V6, phenolic levels increased under R with 4 mL but decreased under S. Anthocyanins did not vary at V3, but at V6, their levels increased dose-dependently under R, and decreased under S (Figure 6B). Flavonoids followed a similar trend, with significant differences between 0 mL in R and S (Figure 6C).

#### 3.1.3. Multivariate Analysis from Physiological and Biochemical Assessments

**Figure 7:**
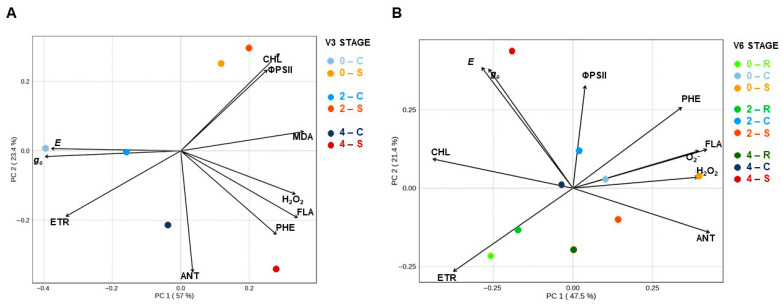
Principal Component Analysis (PCA) between biofertilizer doses (0, 2 and 4 mL kg^−1^ seed) and heat treatment conditions (R = recovery, C = control, S = stress) in soybean plants [*Glycine max* (L.) Merr., cv. Brasmax Valente] at V3 (**A**) and V6 stage (**B**). Biplot between PC1 and PC2 show the contribution of physiological and biochemical traits (*g_s_* = Stomatal conductance, *E* = Transpiration Rate, ΦPSII = Photosystem II Quantum Efficiency, ETR = Electron Transport Rate, CHL = Chlorophyll index, H_2_O_2_ = Hydrogen Peroxide, MDA = Malondialdehyde, O_2_^−^ = Superoxide generation rate, PHE = Phenols, ANT = Anthocyanins and FLA = Flavonoids) in the variability and segregation of soybean plants exposed to different heat treatments. Different colors indicate different doses and heat treatment conditions, as shown in the graph legend.

The Principal Component Analysis (PCA) integrated the physiological and biochemical variables for both V3 (Figure 7A) and V6 (Figure 7B) stages. At V3, PCA explained 80.4% of the total data variance, with PC1 (57%) separating the treatments based on the trade-off between physiological performance and biochemical responses. Secondary metabolites (PHE, FLA, and ANT) and H_2_O_2_ showed a positive correlation with the 4 mL dose under S, suggesting that the higher dose of SME elicited an early mobilization of the protective metabolism. In contrast, the 0 and 2 mL dose under S were positively associated with MDA and chlorophyll index, locating themselves in the upper quadrant, opposite to the control groups characterized by higher physiological traits (*g_s_*, *E*, and ETR).

At V6 stage, PCA explained 68.9% of the total data variance, with PC1 (47.5%) separating, again, the treatments due to physiological traits and oxidative stress. Physiological traits (*g_s_*, *E*, ETR, and CHL) showed a strong positive correlation with the 4 mL dose under S, indicating that the higher dose of SME enhanced physiological performance during thermal stress. In contrast, the 0 and 2 mL dose under stress were positively associated with stress markers such as O_2_^−^ and H_2_O_2_, locating themselves in opposite quadrants to the recovery groups. Furthermore, along the PC1 axis, the 4 mL dose in R clustered closely with the control groups, suggesting that this treatment promoted a more effective restoration of physiological homeostasis.

**Figure 8:**
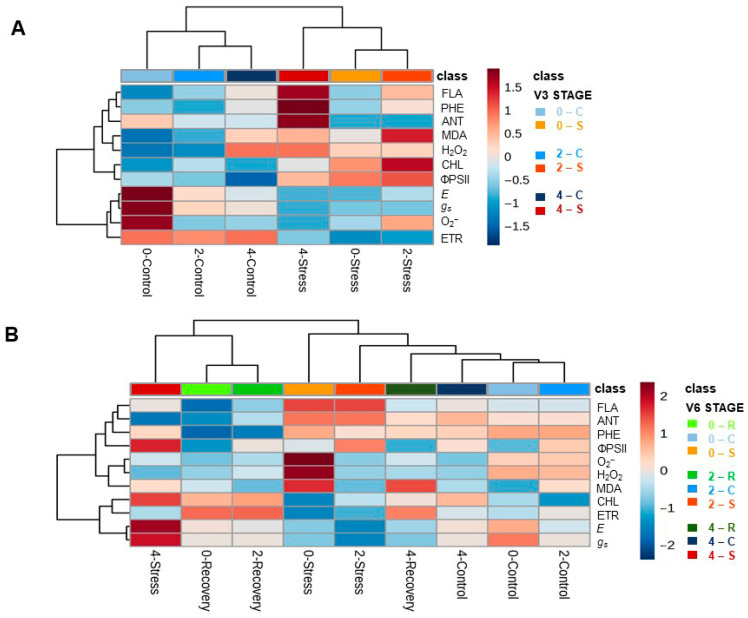
Hierarchical clustering and heatmap analysis of biofertilizer doses (0, 2 and 4 mL kg^−1^ seed) and heat treatment conditions (R = recovery, C = control, S = stress) in soybean plants [*Glycine max* (L.) Merr., cv. Brasmax Valente] at V3 (**A**) and V6 stage (**B**). The heatmap displays the relative changes in physiological and biochemical traits (*g_s_* = Stomatal conductance, *E* = Transpiration Rate, ΦPSII = Photosystem II Quantum Efficiency, ETR = Electron Transport Rate, CHL = Chlorophyll index, H_2_O_2_ = Hydrogen Peroxide, MDA = Malondialdehyde, O_2_^−^ = Superoxide generation rate, PHE = Phenols, ANT = Anthocyanins and FLA = Flavonoids). Color gradients represent the Z-score (standardized values), ranging from blue (downregulation/decrease) to red (upregulation/increase). The dendrograms indicate the similarity between treatments (top) and variables (left) based on Euclidean distance.

The hierarchical clustering based on Euclidean distance and Ward’s linkage integrated all physiological and biochemical variables across treatments in Figure 8. At V3 stage (Figure 8A), the dendrogram revealed a primary separation between C and S treatments. Within the stress groups, the 2 mL dose under S clustered closely with the 0 mL under S treatment. Conversely, the 4 mL dose under S formed a distinct intermediate cluster, exhibiting a profile more similar to the C groups than to the other stress-affected treatments.

The hierarchical grouping of variables established two functional categories that drove this clustering. The first group included oxidative stress markers (MDA and H_2_O_2_), the non-enzymatic antioxidant system (Flavonoids, Phenols, and Anthocyanins), and photosynthetic parameters (chlorophyll and ΦPSII). The second group was primarily defined by gas exchange traits (*g_s_* and *E*), O_2_^−^, and the Electron Transport Rate.

At the V6 stage (Figure 8B), the hierarchical clustering revealed a primary separation based on the plants’ recovery capacity and current physiological status. The recovery groups (R), previously stressed at V3, clustered in close proximity to the C treatments, indicating a return to physiological homeostasis. Among the treatments under active stress, the 2 mL dose exhibited a profile more closely aligned with non-stressed plants (R and C), while the 0 mL dose formed an intermediate cluster. Notably, the 4 mL dose under S formed a distinct group, aligning more closely with the recovery profiles of the 0 and 2 mL doses than with the other active stress treatments.

### 3.2. Experiment 2, Molecular Analysis

#### Gene Expression

**Figure 9:**
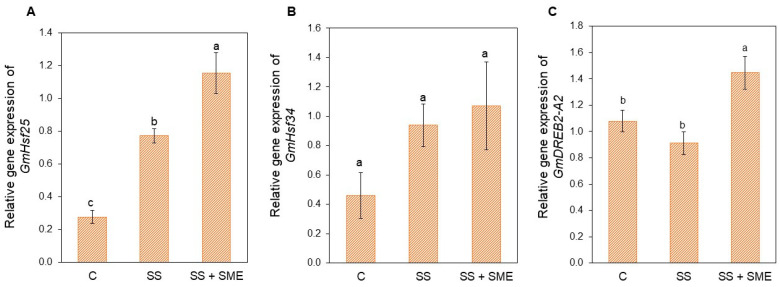
Relative gene expression of *GmHsf-25* (**A**), *GmHsf-34* (**B**) and *GmDREB2-A2* (**C**) in soybean [*Glycine max* (L.) Merr., cv. RGT Symbala] after two consecutive high temperature conditions (at V3 + V6 stages) and SME application. Bars represent means ± standard error (n = 5). Letters indicate significant differences between the different conditions (C = control, SS = stress, SS+SME = stress + SME application). Statistical comparisons were performed using One-way ANOVA followed by Tukey’s post hoc test (*p* ≤ 0.05).

The relative expression level of different genes involved in heat stress tolerance was measured after two consecutive short heat stresses at 34 °C/28 °C for 48 h with or without the application of SME biofertilizer. The two consecutive heat stress applications significantly induced the expression of *GmHsf25* but not that of *GmHsf34* and *GmDREB2-A2* (Figure 9A–C). However, *GmHsf25* and *GmDREB2-A2* were significantly overexpressed in the presence of SME biofertilizer (SS+SME) compared to both control (C) and stressed (SS) plants (Figure 9A–C).

**Figure 10:**
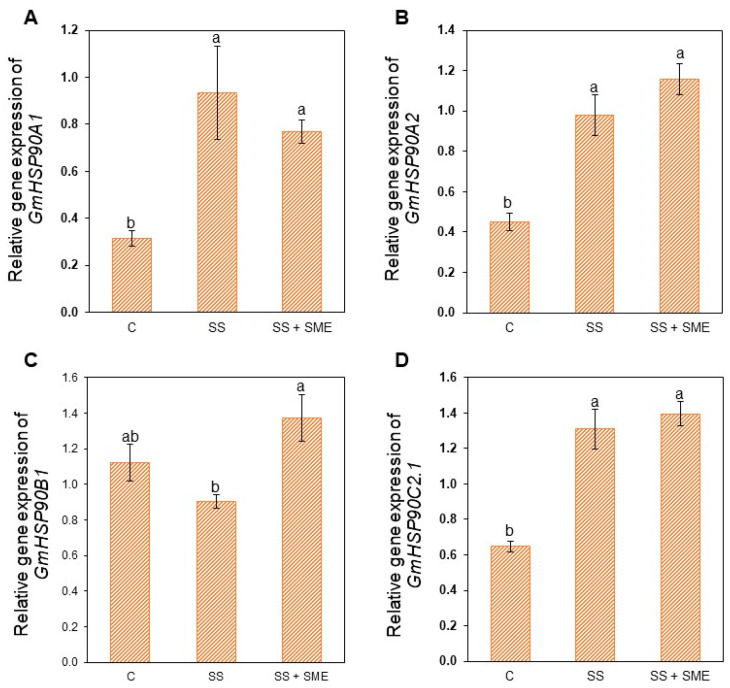
Relative gene expression of *GmHSP90A1* (**A**), *GmHSP90A2* (**B**), *GmHSP90B1* (**C**) and GmHSP90C2.1 (**D**) in soybean [*Glycine max* (L.) Merr., cv. RGT Symbala] after two consecutive high temperature conditions (at V3 + V6 stages) and SME application. Bars represent means ± standard error (n = 5). Letters indicate significant differences between the different conditions (C = control, SS = stress, SS+SME = stress + SME application). Statistical comparisons were performed using One-way ANOVA followed by Tukey’s post hoc test (*p* ≤ 0.05).

The expression level of different *GmHSP90* genes was also measured (Figure 10). Among the four analyzed genes, three were significantly overexpressed under the second heat stress treatment (SS) compared to control plants (*GmHSP90A1*, *GmHSP90A2* and *GmHSP90C2.1*) and one of them (*GmHSP90B1*) was significantly overexpressed in SS+SME plants compared to S plants.

## 4. Discussion

The data obtained in this study provide evidence that the application of the biofertilizer SME as a seed priming agent modulates key physiological and biochemical responses in soybean plants subjected to high-temperature stress at two different developmental stages, V3 and V6. It also influenced gene expression when plants were subjected to heat stress twice. Notably, the 4 mL kg^−1^ dose induced consistent changes in gas exchange parameters, enhanced antioxidant regulation, and elicited memory-like responses at the V6 stage, particularly under stress conditions.

### 4.1. Effects of the SME Seed Treatment as Priming in the Physiological and Biochemical Parameters

Under S, both *g_s_* and *E* declined across all doses, reflecting the expected stomatal closure due to high temperatures at the V3 stage, showing that plants could be more resilient, as they have received a priming effect due to SME seed treatment [14,24].

At V6, there is an enhancement in *g_s_*, *E*, and ETR in plants with 4 mL dose, which may reflect a controlled stomatal opening, balancing evaporative cooling with water loss, a mechanism previously associated with heat tolerance in legumes [4,9]. These findings are in line with prior studies reporting improved physiological resilience in primed plants due to metabolic reprogramming and enhanced cellular signaling pathways, suggesting that SME treatment may have promoted a form of somatic stress memory [14,16,19]. Also, during stress at V6 stage, the highest ETR was detected under R, indicating that prior stress may have triggered a memory effect, especially at 4 mL [16,23].

The differential response between developmental stages (V3 vs. V6) reinforces the hypothesis that priming effectiveness is stage dependent. At earlier stages (V3), physiological changes were less pronounced and more variable, suggesting that the cellular machinery required for memory acquisition may not yet be fully developed or responsive [11,38]. In contrast, V6 plants exposed to priming as seed treatment exhibited physiological profiles similar to non-stressed controls, indicating a degree of acclimation or retained information from the priming stimulus.

Biochemical analyses further support this hypothesis. The reduced accumulation or production of reactive oxygen species (H_2_O_2_ and O_2_^−^, respectively) in plants treated with SME under heat stress suggests improved oxidative homeostasis, likely due to primed activation of antioxidant pathways; the limited MDA variation reinforces that oxidative damage was minimal and that antioxidant systems were efficient in mitigating lipid peroxidation [3,10]. Notably, these changes occurred without a proportional increase in non-enzymatic antioxidants such as phenolics, Flavonoids, or Anthocyanins at V6, implying a shift toward more efficient, possibly pre-activated enzymatic defense systems [8,39].

### 4.2. Multivariate Analysis Reveals Stage-Dependent Metabolic and Physiological Grouping

The metabolic divergence observed in the PCAs between the V3 and V6 stages suggests that the seed application of SME induces a temporal reprogramming of heat tolerance strategies. At the V3 stage, the association of the 4 mL dose with non-enzymatic antioxidants and H_2_O_2_ indicates a stress signaling phase and the initiation of biochemical priming [3,40]. This early response is characteristic of a state of metabolic readiness, where the plant prioritizes photoprotection and membrane stabilization over immediate biomass accumulation, thereby preparing the photosynthetic apparatus for subsequent or persistent thermal challenges [41,42].

This biochemical preparation at V3 is directly reflected in the physiological resilience observed at V6. While plants without SME (0 mL dose) remained stagnant in an oxidative stress profile (O_2_^−^ and H_2_O_2_), the plants treated with 2 mL dose under S appear close to control groups and 4 mL dose under S migrated toward a quadrant of high physiological performance (*g_s_*, *E*, and ETR). This shift suggests the establishment of somatic stress memory, enabling the plant to maintain gas exchange homeostasis even under adverse conditions [15,19]. This pattern aligns with findings in rice and *Arabidopsis* where priming induced persistent transcriptional and metabolic alterations that contributed to stress tolerance [18,20,43].

The proximity of the 4 mL under R to the control groups at the end of the experiment indicates that primed plants not only withstand stress more effectively but also recover faster, a hallmark of priming efficacy [15,44].

Moreover, the dose-dependent responses observed highlight the importance of precise calibration when applying SME. The 4 mL dose elicited more robust physiological changes compared to 2 mL in some cases, especially under R conditions. These results are consistent with the non-linear nature of plant responses to growth regulators and the biofertilizer, as previously described [27,45].

The hierarchical clustering analysis across the V3 and V6 stages reinforces that seed application of SME triggers a temporal reprogramming of heat tolerance. At the V3 stage, the formation of a distinct intermediate cluster for the 4 mL dose under S, positioned closer to the control groups than to the other stress treatments (0 and 2 mL), suggests an early mitigation of heat-induced damage. This clustering pattern is primarily driven by the modulation of H_2_O_2_ and the non-enzymatic antioxidant system (Phenols, Flavonoids, and Anthocyanins). The association of the higher dose with these markers indicates that, instead of suffering from oxidative degradation, the plants treated with 4 mL utilized H_2_O_2_ as a signaling molecule to initiate biochemical priming, establishing a state of metabolic readiness [19,46,47].

This early investment in protective metabolism at V3 is a key factor for the homeostasis observed at the V6 stage. The dendrogram at V6 clearly separates the plants based on their current physiological status, where the recovery groups (R) clustered in close proximity to the control, demonstrating a successful return to homeostasis [18]. Notably, the 4 mL dose under S (active stress) did not cluster with the other stressed treatments (0 and 2 mL); instead, it aligned more closely with the recovery profiles. This shift in Euclidean proximity indicates that the 4 mL dose promoted a metabolic state that parallels a recovery phase even while under active thermal challenge, effectively minimizing the impact of the stressor on gas exchange and the Electron Transport Rate [11].

The divergence between the 2 mL and 4 mL doses under S throughout the experiment highlights a clear dose-dependent response in the establishment of stress memory. While the 2 mL dose remained closer to the 0 mL stress profile at V3, the 4 mL dose consistently occupied a distinct metabolic space, prioritizing membrane stabilization and the antioxidant machinery [48]. The proximity of the 4 mL treatments to the control groups at the end of the experiment serves as a hallmark of priming efficacy, suggesting that the higher dose not only prepares the photosynthetic apparatus for persistent challenges but also accelerates the transition from a stress-response state to physiological recovery [19].

### 4.3. Effects of the SME Seed Treatment on Gene Expression After Two Heat-Stress Imposing

As a complementary line of evidence, we provide an independent gene expression assay under repeated heat stress. Because this experiment was conducted using a distinct protocol and another soybean cultivar, these results should be interpreted strictly as exploratory molecular consistency, rather than mechanistic validation of the main findings. Still, the transcriptional patterns observed after repeated heat stress are consistent with the priming-like framework discussed in this study. Notably, SME seed exposure was associated with increased expression of key heat-responsive regulators (e.g., *GmHsf25* and *GmDREB2-A2*) relative to stressed controls (Figure 9), a response that plausibly aligns with improved photochemical performance and reduced oxidative burden under heat stress [49,50].

The expression level of different *GmHSP90* genes showed us the benefits of recurrent heat stress when SME was applied as priming effector as seed treatment, since these genes are major players in the tolerance to heat stress [51,52]. Together, these molecular data strengthen the biological plausibility that SME treatment can modulate stress-responsive regulatory pathways in a manner compatible with acquired tolerance under recurrent heat episodes. Also, the upregulation of *GmHSP90B1* by SME application aligns with the role of HSP90 as a key player in extending thermomemory duration, as its sustained accumulation is essential for plant resilience during the recovery phase [53,54]. This is coupled with the reality of the field cultivation where heat waves can occur even more than twice during a plant’s cultivation according to each region, and with the increase in climatic change’s extreme events.

## 5. Conclusions

**Figure 11:**
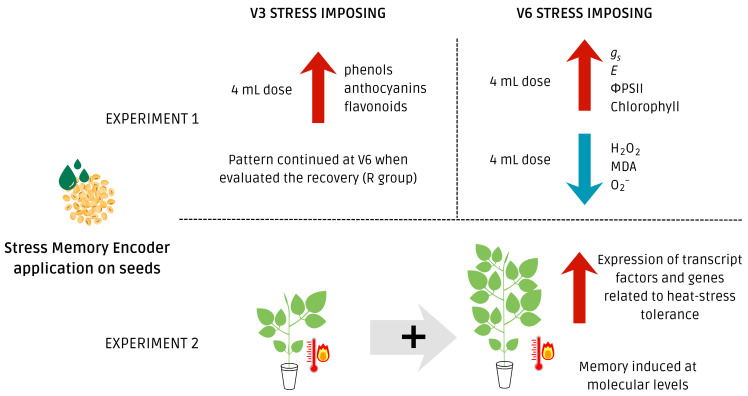
Schematic view of the beneficial effects of the application of SME as a priming effector in soybean plants. Plant memory refers to the ability of plants to retain information from previous stimuli and use it to modify future responses. Prior exposure to a priming effector can induce a primed state, enabling faster, stronger, or more efficient physiological and molecular responses upon subsequent challenges. This memory is mediated by persistent changes in signaling networks, gene expression, and metabolic pathways, allowing plants to optimize performance under recurring or future stress conditions [11]. Red arrows indicate increments, while blue arrows indicate decreases in the studied variables. Gray arrow indicates an addition, as for stress applied twice on those plants, at V3 and V6 stage.

Taken together, the findings of this study demonstrate that SME can modulate physiological, biochemical, and gene expression responses in soybeans in a manner consistent with the induction of somatic stress memory (Figure 11). The modulation of photosynthesis, gas exchange, and oxidative metabolism indicates that SME may be a viable tool for enhancing thermal stress tolerance. The gene expression results also contribute to the somatic memory, regarding its methodology differences, once we integrate its results as a prior time and space scale analysis, which may be implied in the observed physiological and biochemical aspects of plants’ sessile lifestyle, regarding the methodological differences [55,56]. Further investigations under field conditions, especially involving combined stresses and productivity metrics, are warranted to better define the agronomic implications and optimize application strategies.

Stress Memory Encoder (SME) demonstrated a stronger memory effect on physiological responses to heat stress (35 °C for 48 h). Particularly, at V6 under stress, the 4 mL dose increased *g_s_* and *E*, leading to higher ΦPSII and ETR. These improvements were accompanied by reduced H_2_O_2_ and O_2_^−^, with no corresponding increase in non-enzymatic antioxidants, indicating efficient oxidative mitigation without stimulating phenolics, Flavonoids, or Anthocyanins. Besides that, the increased expression of thermotolerant genes as well demonstrates that seed priming with SME can alleviate the heat-stress effects on its perceiving receptors and transcription factors subsequently.

## Data Availability

Data may be available when requested.
